# The development of an intermediate‐duration tag to characterize the diving behavior of large whales

**DOI:** 10.1002/ece3.2649

**Published:** 2016-12-20

**Authors:** Bruce R. Mate, Ladd M. Irvine, Daniel M. Palacios

**Affiliations:** ^1^ Marine Mammal Institute and Department of Fisheries and WildlifeOregon State UniversityNewportORUSA

**Keywords:** animal‐borne data loggers, archival whale tags, biologging, diving and foraging behavior, Fastloc GPS

## Abstract

The development of high‐resolution archival tag technologies has revolutionized our understanding of diving behavior in marine taxa such as sharks, turtles, and seals during their wide‐ranging movements. However, similar applications for large whales have lagged behind due to the difficulty of keeping tags on the animals for extended periods of time. Here, we present a novel configuration of a transdermally attached biologging device called the Advanced Dive Behavior (ADB) tag. The ADB tag contains sensors that record hydrostatic pressure, three‐axis accelerometers, magnetometers, water temperature, and light level, all sampled at 1 Hz. The ADB tag also collects Fastloc GPS locations and can send dive summary data through Service Argos, while staying attached to a whale for typical periods of 3–7 weeks before releasing for recovery and subsequent data download. ADB tags were deployed on sperm whales (*Physeter macrocephalus; N* = 46), blue whales (*Balaenoptera musculus; N* = 8), and fin whales (*B. physalus; N* = 5) from 2007 to 2015, resulting in attachment durations from 0 to 49.6 days, and recording 31 to 2,539 GPS locations and 27 to 2,918 dives per deployment. Archived dive profiles matched well with published dive shapes of each species from short‐term records. For blue and fin whales, feeding lunges were detected using peaks in accelerometer data and matched corresponding vertical excursions in the depth record. In sperm whales, rapid orientation changes in the accelerometer data, often during the bottom phase of dives, were likely related to prey pursuit, representing a relative measure of foraging effort. Sperm whales were documented repeatedly diving to, and likely foraging along, the seafloor. Data from the temperature sensor described the vertical structure of the water column in all three species, extending from the surface to depths >1,600 m. In addition to providing information needed to construct multiweek time budgets, the ADB tag is well suited to studying the effects of anthropogenic sound on whales by allowing for pre‐ and post‐exposure monitoring of the whale's dive behavior. This tag begins to bridge the gap between existing long‐duration but low‐data throughput tags, and short‐duration, high‐resolution data loggers.

## 1 Introduction

Understanding how animals use their environment at multiple scales is a key goal in behavioral ecology. Data loggers and tracking devices have been used in various forms for over 30 years to monitor animal activity during times when they cannot be observed (Cooke et al., 2004; Mate, Mesecar, & Lagerquist, 2007; Ropert‐Coudert & Wilson, 2005). This type of remote monitoring is especially valuable in the field of marine mammal research because the study subjects spend the majority of their time below the surface of the water, where direct observation ranges from very difficult to impossible. Researchers working with pinniped species have had great success attaching data loggers to their subjects’ pelage in order to study movement, diving physiology, and body condition over periods of months (Biuw, McConnell, Bradshaw, Burton, & Fedak, 2003; Costa & Gales, 2003; Guinet et al., 2014). Large‐whale researchers have faced a much greater challenge due to the impossibility of capturing or otherwise controlling the subject during tag attachment. Two main types of tag attachment are currently used to study whales, each with advantages and disadvantages. Transdermal attachments have been used with increasing regularity for satellite‐monitored tags since the mid‐1990s to document long‐term (months) movements (Andrews, Pitman, & Ballance, 2008; Heide‐Jorgensen, Witting, & Jensen, 2003; Mate et al., 2007). Some of these tags can now function for over one year (Mate et al., 2007); however, their data can only be recovered through the Argos satellite system, which drastically limits the amount of information that can be transmitted. On the other hand, suction‐cup‐attached data loggers are capable of recording dive depth, body orientation, and acoustic data at rates >16 Hz (Burgess, 2000; Johnson & Tyack, 2003), but the large quantities of data generated cannot be sent via satellite, so they are stored on board for download after the tag is recovered, which typically occurs within 24 hr of deployment on large cetaceans (Fais et al., 2015; Simon, Johnson, & Madsen, 2012), with occasional longer deployments reported (34 h: Goldbogen, Calambokidis et al., 2013; 62 h: Amano & Yoshioka, 2003).

While high‐resolution data loggers can record relatively large amounts of information on dive behavior, they cannot be used to characterize how that behavior changes over time due to the short‐attachment duration. This knowledge gap represents the next frontier in technology development for whale research, particularly in the face of the growing need to document how these sensitive species might respond to various sources of anthropogenic disturbance such as noise from commercial vessel traffic, mid‐frequency naval sonar, or seismic exploration vessels (Nowacek, Thorne, Johnston, & Tyack, 2007; Soto et al., 2006; Southall et al., 2007) beyond short‐term responses (DeRuiter et al., 2013; Goldbogen, Calambokidis et al., 2013; Goldbogen, Southall et al., 2013). In order to better understand whale behavior over a longer temporal scale, and to identify behavioral changes that may result from exposure to anthropogenic noise, a high‐resolution data logger is needed that can stay attached to a whale for periods of several weeks or more (Johnson, Tyack, Gillespie, & McConnell, 2013; Nowacek, Christiansen, Bejder, Goldbogen, & Friedlaender, 2016).

Earlier attempts to study whale diving behavior with longer‐duration tags have been made with some success (Baumgartner, Hammar, & Robbins, 2015; Davis et al., 2007; Schorr, Falcone, Moretti, & Andrews, 2014), although the resolution and types of data collected have remained inferior to those obtained from short‐duration, high‐resolution data loggers. Recent development of methods for longer attachment of high‐resolution data‐logging packages has provided records for 7.6–16 days (Owen, Jenner, Jenner, & Andrews, 2016; Szesciorka, Calambokidis, & Harvey, 2016), representing progress toward this goal and showing the utility of high‐resolution data collected over multiple days.

Here, we describe the development through four generations of the Advanced Dive Behavior (ADB) tag, a spatially explicit, high‐resolution (1‐Hz) data logger for large whales capable of staying attached for intermediate time periods (weeks to >1 month). The design focused on a semi‐implantable style that allowed the tag to record data onboard, then release from an attachment housing and float to the surface for subsequent recovery and data download. The greater attachment duration and ability to set a release date for recovery are a significant step toward the goal of measuring large whale behavior. The data records obtained from this tag will dramatically advance our understanding of cetacean ecology.

## 2 Materials and Methods

### 2.1 Tag configuration and deployment

**Figure 1:**
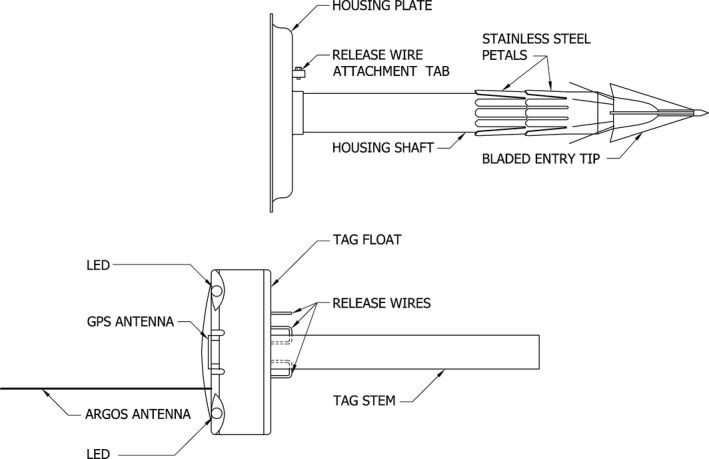
A schematic drawing of the external design of the ADB tag (bottom) with the deployment housing (top)

The ADB tag is a novel configuration of the Wildlife Computers (Seattle, Washington, USA) Mk10‐PATF pop‐up archival time‐depth‐recorder tag. The external design of the ADB tag is shown schematically in Figure 1. The basic design across all four generations included control boards for the sensors and two lithium batteries cast into a tube affixed to an 8.75‐cm‐diameter syntactic foam float. A Fastloc GPS patch antenna (Bryant, 2007) was incorporated into the top of the float along with an Argos antenna, LED lights for recovery, a hydrostatic pressure sensor, and a saltwater conductivity sensor to detect surfacing events. A corrodible link wire in the form of a loop was mounted to the underside of the float for attachment to the deployment housing until the desired release time was reached. Generation‐3 and Generation‐4 ADB tags were equipped with three release wires so they could be redeployed after recovery. Tags were also equipped with light level and temperature sensors as part of the original Mk10‐PATF configuration. Three‐axis accelerometers were incorporated into the tags in Generation 2, while magnetometers and a Fastloc‐3 GPS receiver were added in Generation 3 (Table 1).

**Table 1:** Summary of components included in four generations of ADB tags

Sensor (Resolution)	Generation 1	Generation 2	Generation 3	Generation 4
Stem Dimensions (cm)	2 × 15	2 × 11.5	2 × 11.5	2 × 11.5
Fastloc version	v. 1	v. 1	v. 1	v. 3
Depth (±0.5 m)	Yes	Yes	Yes	Yes
Three‐Axis Accelerometers (±0.05 G)	No	Yes	Yes	Yes
Three‐Axis Magnetometers	No	No	Yes	Yes
Temperature (±0.05°C)	Internal	External	External	External
Light Level	Internal	External	External	External
Release Wires	1	1	3	3

For deployment, the tag was inserted into an attachment housing constructed from 16‐gauge surgical‐quality 316 stainless steel consisting of an 18.5‐ or 14.5‐cm‐long, 2.6‐cm‐diameter shaft (Generation 1 and generations 2‐4, respectively) affixed to a plate with a raised lip to protect the foam float (Figure 1). Four cutting blades attached to a Delrin nose cone were affixed to the distal end of the shaft, along with two rows of backward facing petals in a similar configuration to implantable tags described in Mate et al. (2007). The tag was secured to the housing by threading a small screw through the release wire and then through a perpendicular tab below the housing plate.

For deployment, tags were attached to a carrier (a “pushrod”) using a sculpted Delrin attachment that held the outside of the float with pressure provided by an O‐ring while it applied force to the rim of the attachment housing plate. A tag and pushrod were deployed at close range (2–4 m) from a 6.8‐m rigid‐hulled inflatable boat using the Air Rocket Transmitter System, a modified line‐thrower using compressed air (Heide‐Jorgensen et al., 2003; Mate et al., 2007), charged to 125 psi (for blue whales, *Balaenoptera musculus* and fin whales *B. physalus*) or 140 psi (for sperm whales, *Physeter macrocephalus*). Tags were deployed 0.25–4 m forward of the dorsal fin/hump of the whale, depending on the species, and no more than 20 cm down from the midline. Care was taken to place the tag perpendicular to the surface of the whale, so the plate and float would sit flat to minimize drag. The impact of deployment separated the pushrod from the tag for recovery.

### 2.2 Data collection and transmission

All collected data were stored in an onboard archive, and the complete data record could only be accessed by recovering the tag for download. ADB tags were programmed to collect sensor data (depth, light level, temperature, accelerometers, and magnetometers) at 1 Hz for the duration of all deployments. Collection of Fastloc GPS locations could occur at regular, user‐specified intervals (i.e., 1 location per hour), or immediately after the whale surfaced from a “qualifying dive” defined by the user.

Argos messages were transmitted every 45 s while the whale was at the surface. A saltwater conductivity switch prevented transmissions while the tag was underwater. An Argos transmission could contain (1) a location message containing one set of Fastloc GPS pseudo‐ranges; (2) one of four types of histogram summary messages for qualifying dives (time at depth, time at temperature, maximum dive depth, and dive duration); (3) a behavior message with summaries of four consecutive qualifying dives listing the dive date/time, maximum dive depth, dive duration, dive shape, and subsequent surfacing duration; or (4) a utility message summarizing battery voltage, number of Argos transmissions, and number of Fastloc attempts. The Argos messages could be assigned differing priorities and allowed same‐day monitoring of the whale's diving behavior and location while the tag was attached.

### 2.3 Programmed release and recovery

Release from the tag housing could be triggered by three possible criteria: Reaching a user‐specified release date and time, if the estimated remaining battery life of the tag was reduced to one‐fourth capacity, or if the tag recorded, a constant depth (±10 m) for 24 h, indicating the tag and housing were shed from the whale and sank prior to scheduled release. Tag release was identified by a change in Argos transmission interval from every 45 s to once per minute. Postrelease, new Fastloc GPS locations were acquired hourly to aid with recovery. When a release was identified, recent locations were downloaded from Argos to define an initial search area and direction of drift for the tag. An uplink receiver and accompanying software on a computer carried onboard the recovery vessel were capable of receiving, decoding, and solving location messages sent by the tags at a range of ≤3 nautical miles. Solved locations from this system were used to focus the search area within 50 m of the floating tag, so that it could be located visually from the vessel. The three LED lights on the float made tags easier to locate at night.

### 2.4 Predeployment accuracy testing

In 2007, four Generation‐1 ADB tags were affixed to a life ring at a variety of angles and allowed to drift on the water for 90 min with the tags set to collect Fastloc GPS locations every 5 min. The results of those locations were compared to locations collected by a Garmin‐72 GPS unit that was also affixed to the life ring to assess the accuracy of ADB‐generated GPS locations.

## 3 Results

### 3.1 Deployment information, attachment duration, and tag recovery

Advanced Dive Behavior tags were first deployed on sperm whales in the central Gulf of California, Mexico, during March–June 2007 and 2008. Attachment duration ranged from 0.5 to 34.5 days (Table 2; Table S1) but would have been longer in some cases, as seven tags released early in 2007 and an epoxy casting defect that left air bubbles near key components likely caused an unknown number deployed in 2008 to fail prior to release (see Complications with attachment and release). During that time, the recovered tags archived 31–1,183 dives and 31–850 Fastloc GPS locations (Table 2; Table S1). Generation‐2 ADB tags were deployed on 11 sperm whales in the northern Gulf of Mexico, USA, during summer 2011, and nine Generation‐3 ADB tags were deployed in the same area during 2013. Median attachment duration was 25.3 days (range: 9.6–49.6 days) in 2011 and 16.7 days (range: 0–24.9 days) in 2013 (Table 2; Table S1). Recovered tags archived 27–1,111 dives and 59–1,355 Fastloc GPS locations (Table 2; Table S1).

**Table 2:** Summary of ADB tag deployment and archived data

Year	Species	ADB tag type	Duration (days)	No. archived GPS locations	No. archived dives	No. recovered
2007, 2008	Sperm	G1 (*n* = 26)	2.4 (0.5–34.5)	215 (31–850)	214 (31–1,183)	10
2011	Sperm	G2 (*n* = 11)	25.3 (9.6–49.6)	666	1,111	1
2013	Sperm	G3 (*n* = 9)	16.7 (0–24.9)	758 (59–1,355)	390 (27–671)	7
2014, 2015	Blue	G3 (*n* = 3)	19.8 (19.0‐ 24.8)	799 (185–1,558)	2,075 (1,068–2,918)	3
2014	Fin	G3 (*n* = 3)	13.3 (4.9–15.8)	NA (95–221)	NA (343–1,140)	2
2014, 2015	Blue	G4 (*n* = 5)	25.9 (18.3–28.9)	2,317 (1,480–2,539)	2,278 (2,075–2,794)	4
2015	Fin	G4 (*n* = 2)	15.7 (15.4–16.0)	1,591	910	1

Median values and range are listed. See Table S1 for individual tag data. Any submergence >10 m depth and 1 min duration was counted as a dive.

Generation‐3 and Generation‐4 ADB tags were deployed on both blue whales and fin whales off southern California, USA, during summer 2014–2015. Median attachment duration for the tagged blue whales was 19.8 days (Generation 3, *n* = 3) and 25.9 days (Generation 4, *n* = 5; range across both generations: 18.3–29.8 days; Table 2; Table S1), with all but two reaching or exceeding their programmed release date (see Complications with attachment and release). Median attachment duration for the tagged fin whales was 13.3 days (Generation 3, *n* = 3) and 15.7 days (Generation 4, *n* = 2; range across both generations: 4.9–16.0 days), with two tags reaching their programmed release date. Recovered tags archived 1,068–2,918 dives and 185–2,539 Fastloc GPS locations for blue whales, and 343–1,140 dives and 95–1,591 Fastloc GPS locations for fin whales across both generations (Table 2; Table S1).

Tag recovery was complicated by the extended attachment duration of the tags, which allowed some tagged whales to travel >500 km from the tagging area before tag release. In such cases, it was most economical to charter a local vessel from the closest port to attempt recovery. Poor weather and the tag's distance from shore (>160 km in some cases) were further limitations to recovery, such as tags 2013_5701 and 2015_5744 that continued transmitting until their batteries were exhausted. Tag 2013_5701 was found >1 year later by beachgoers and returned, as were three others in different years, demonstrating the continuing possibility of tag recovery following field work.

### 3.2 Assessment of Fastloc GPS location accuracy

**Figure 2:**
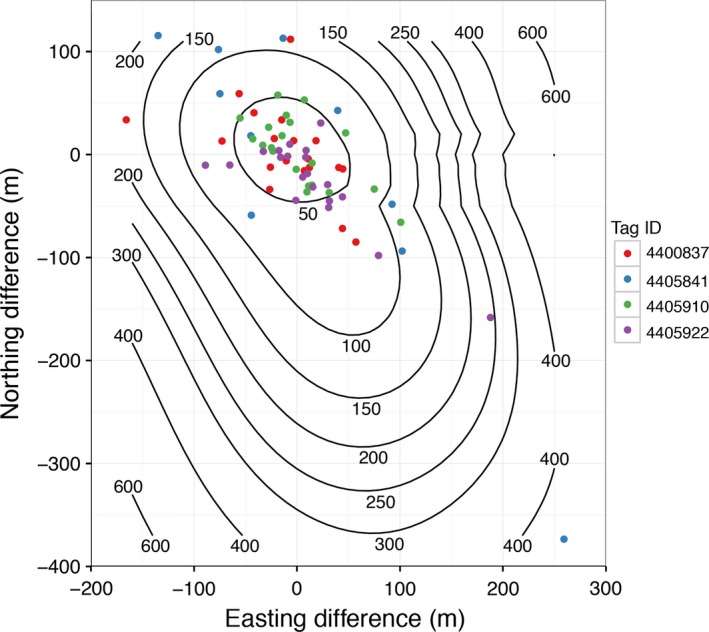
The easting and northing components of the distance between ADB tag Fastloc GPS locations and a handheld GPS unit for four Generation‐1 tags for which accuracy testing was conducted, showing a slight bias in the northwest–southeast direction for all tags and larger errors for tag # 4405841, as discussed in the text. Contours represent the straight‐line distance (in m) between Fastloc and handheld GPS locations, interpolated over the easting and northing differences

In 2007, predeployment testing using four tags showed that the median straight‐line distance between a handheld GPS location and a Fastloc GPS location collected by the tags was 43 m, 83% of distances were less than 100 m and all distances were less than 455 m. Distance decreased with increasing number of satellites recorded, as has been observed in other studies (Dujon, Lindstrom, & Hays, 2014; Hazel, 2009). In general, distances were normally distributed in both the easting and northing directions, although there was a slight bias in the northwest–southeast direction (Figure 2). The root‐mean‐square error (RMSE) of these distances for all tags was 92.2 m, but RMSE from one tag (# 4405841) was over twice that of the other three (RMSE = 178.2 m vs. 69.9, 50.2, and 73.6 m). That tag produced all of the locations with only 4 or 5 satellites during the test, which is indicative of poor‐quality locations (Dujon et al., 2014; Hazel, 2009), and only produced half the number of locations as each of the other three tags.

In addition to the number of satellites, Fastloc GPS locations provided a “residual value,” which indicates the relative spatial accuracy of a location. In other studies, locations with residual values greater than 30 have been excluded (Shimada, Jones, Limpus, & Hamann, 2012; Witt et al., 2010), but in our test, only four locations exceeded this threshold (range = 33.7–39.8) and all were <47 m from the true location. All four locations were also associated with a large number of satellites (≥8), suggesting that the identification of poor‐quality Fastloc GPS locations is more complex than indicated by the residual value and/or the number of satellites.

### 3.3 Assessment of tag functionality

#### 3.3.1 Depth data

**Figure 3:**
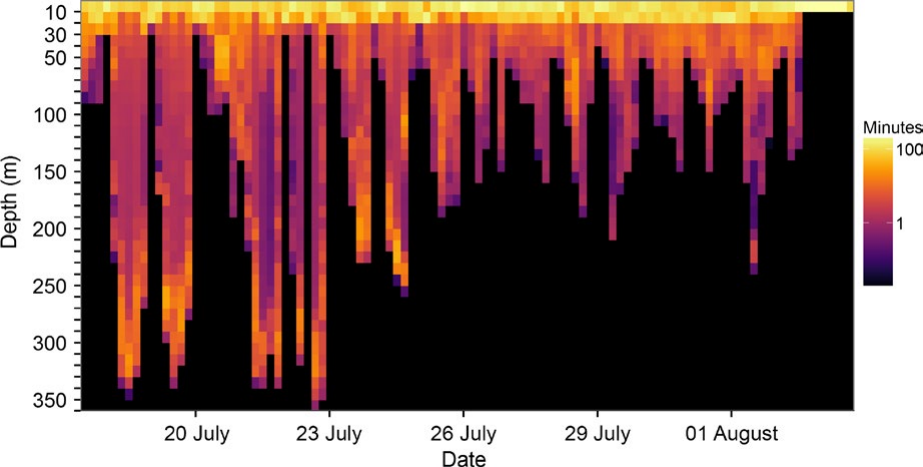
Time spent at depth for an ADB‐tagged fin whale (tag # 2015_5654) tracked off California for 16 days in July 2015. Depth data, sampled at 1 Hz, were divided into 10‐m depth increments and then summed across 4‐hr time intervals. A strong diel pattern is evident throughout the record, with time spent at depth during the day and nighttime periods restricted to the upper 50 m. Over the course of the record, this animal switched from intense deep‐diving (>200 m) activity through 25 July to shallower diving for the remainder of the tracking period

Archived dive profiles from recovered tags were similar to those published for sperm whales (Amano & Yoshioka, 2003; Miller, Johnson, & Tyack, 2004), blue whales, and fin whales (Croll, Acevedo‐Gutierrez, Tershy, & Urbán‐Ramírez, 2001). Blue and fin whale dive profiles often recorded stereotypical upward excursions during the bottom phase of the dive, which are known to indicate feeding lunges (Calambokidis et al., 2007; Croll et al., 2001). Diel variability in dive depths was recorded with deeper dives occurring during the day (Figure 3), and consecutive dives often ascended or descended near sunset or sunrise, respectively, indicating the whales were following vertically migrating prey layers (Calambokidis et al., 2007).

**Figure 4:**
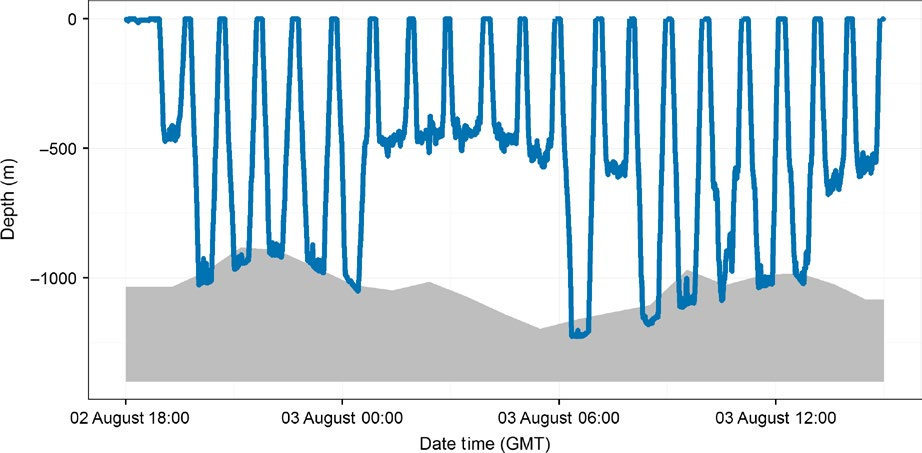
A 20‐h dive profile from ADB tag 2013_840 attached to a sperm whale in the Gulf of Mexico in 2013. The solid gray polygon shows the seafloor depth (from GMRT) nearest to the tag's Fastloc GPS location at the beginning of each dive. Note that several dives reach past the reported seafloor depth, as discussed in the text

While many archived sperm whale dives had similar characteristics to documented pelagic foraging dives (Miller et al., 2004; Watwood, Miller, Johnson, Madsen, & Tyack, 2006), some dives from both the Gulf of California and Gulf of Mexico were recorded to depths >1,600 m. Portions of the dive record during the bottom phase of deep dives showed very little vertical variability and instead changed depth gradually, with the bottom of a subsequent dive beginning close to the depth where the prior dive had stopped (Figure 4). The depths of these dives matched water depths very closely and had the appearance of the whale following, and likely foraging along, the seafloor.

Sperm whales were repeatedly recorded diving deeper than the seafloor depth reported for the dive location (*n* = 458 of 2871 dives in the Gulf of California; *n* = 521 of 2648 dives in the Gulf of Mexico) in the Global Multi‐Resolution Topography Synthesis (GMRT) bathymetric product (Ryan et al., 2009). It is not unexpected that some dives might exceed the reported seafloor depth, as the tags cannot discern the whale's location during a dive, and in many cases, the dive location was on the edge of a steep bathymetric feature. However, some dives, particularly in the Gulf of California, exceeded the listed depth by as much as 200–500 m in areas where bathymetric data show little variability in bottom relief. The GMRT data set has a nominal resolution of 400 m, although it is a combination of available bathymetry data sets at a range of resolutions. The density of the underlying data is a key factor in the accuracy of bathymetric data sets (Marks & Smith, 2006), and high‐resolution bathymetry data from some parts of the Gulf of California are sparse. ADB‐recorded dives exceeding the listed seafloor depth could therefore help improve bathymetric data sets in regions lacking detailed sounding or multibeam survey data, as has been carried out with seals (Padman et al., 2010).

#### 3.3.2 Accelerometer data

**Figure 5:**
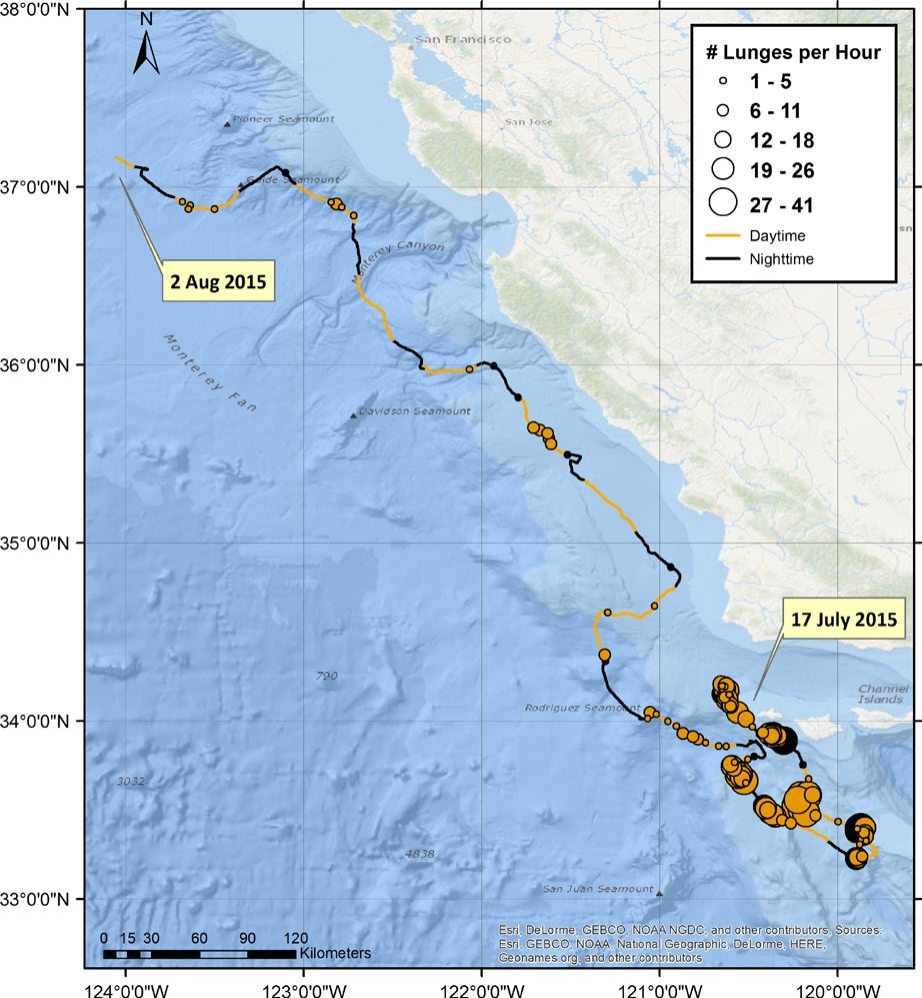
The track of a fin whale (tag # 2015_5654) tracked with an ADB tag off Southern California in July 2015. Circle diameter represents the number of feeding lunges recorded by the tag per hour. The circle is centered on the portion of the track that was summarized

Accelerometer‐derived metrics, such as the “jerk” (the difference in the norm of all three acceleration vectors after removing gravity), can detect rapid changes in orientation and acceleration of a tagged whale associated with foraging events (Miller et al., 2004; Simon et al., 2012). Peaks in ADB‐derived jerks from blue and fin whales showed a close correspondence with stereotypical upward excursions in the depth profile during the bottom portion of a dive, which were previously known to be indicative of a baleen whale lunge‐feeding event (Acevedo‐Gutierrez, Croll, & Tershey, 2002; Goldbogen et al., 2006). By documenting the location and frequency of jerk events across a multiweek track, the ADB tag was able to identify localized areas of high foraging effort (Figure 5) and the depths at which it occurred (Figure 3).

While many current data loggers use a substantially higher sampling rate, the 1‐Hz sampling rate of the ADB tag was sufficient to detect lunges and has been used in past studies that examined baleen whale foraging behavior (Acevedo‐Gutierrez, Croll & Tershy 2002; Goldbogen et al., 2006). More detailed analyses of baleen whale behavior, like the fluking frequency of fin whales, has been examined using a 1‐Hz sampling rate (Goldbogen et al., 2006) and should therefore also be possible with the ADB data from blue and fin whales. However, the fluking rate of smaller species like humpback whales (*Megaptera novaeangliae*) has been shown to be higher than that of fin whales (up to 0.5 Hz; Simon et al., 2012) and confounding factors like aliasing therefore become more problematic as the size of the study animal decreases and the maximum rate of the signal approaches the sampling rate. Accelerometer data from ADB tags sampled at 1 Hz are therefore best suited to examining low‐frequency signals and care should be taken, or a higher sampling rate should be used, when studying smaller species or attempting to examine higher‐frequency signals.

Rapid orientation changes from jerk events also were detected in sperm whale ADB records using accelerometers, predominantly during the bottom phase of dives. Increased rates of orientation change during the bottom phase of a dive have been linked to foraging in sperm whales (Aoki et al., 2012; Miller et al., 2004), although their application is less direct compared to baleen whales. In sperm whales, a prey capture attempt is more reliably distinguished acoustically by a rapid clicking (the “buzz,” detectable by a hydrophone on the tag), which occurs at close range to the prey (Miller et al., 2004). Multiple rapid orientation changes at varying intensities might occur during a pursuit prior to prey capture so, without an onboard hydrophone, the number of ADB‐detected jerk events is not a direct measure of the number of prey capture attempts by the whale during a dive. However, because animals are predicted to forage more intensely in areas of higher prey density (Krebs, 1978; Schoener, 1971), the number of jerk events recorded should be dependent on the number of actual foraging attempts made during a dive. In such a case, the number of jerk events would be a relative measure of foraging effort made by the whale per dive, allowing for the spatial variability of foraging to be examined.

#### 3.3.3 Temperature data

**Figure 6:**
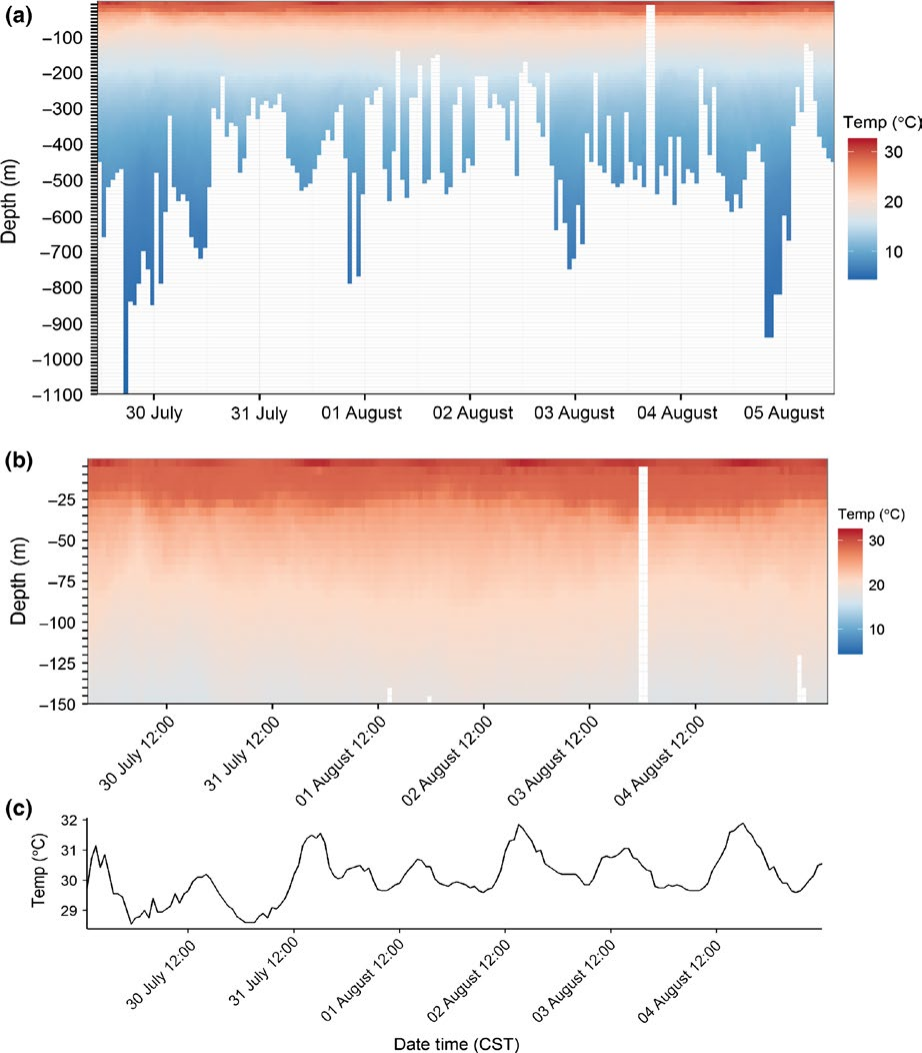
A 1‐week time series of water temperature data collected by an ADB‐tagged sperm whale (tag # 2013_5640) tracked in the northern Gulf of Mexico during August 2013. (a) Average temperature calculated for 10‐m by 1‐hr bins across the entire record showing thermal structure of the water column, including the thermocline in the upper 200 m. (b) A close‐up view of the top 150 m of Figure 5a summarized in 5‐m by 1‐hr bins to better resolve fluctuations in thermocline depth over time. (c) Average temperature of the top 5 m in 1‐hr bins for the same record, showing the daily heating and cooling cycle of surface waters

Generation‐2 through Generation‐4 ADB tags were equipped with external temperature probes capable of sampling the water temperature during a dive. While whales do not make completely vertical dives, and there may be small‐scale differences in the thermal structure of the water column between where they start and end a dive, the measurements are adequate to identify important aspects of the ocean's thermal regime such as the thermocline, or the daily heating of surface waters, with reasonable accuracy (Figure 6). Similar sensors have been used to monitor thermal properties of the ocean in under‐sampled regions (McMahon et al., 2005). Whale‐borne temperature measurements might serve a similar purpose, while also serving to expand habitat models that use satellite‐derived variables like sea surface temperature (Pirotta, Matthiopoulos, MacKenzie, Scott‐Hayward, & Rendell, 2011).

#### 3.3.4 Argos dive summary transmissions

The amount of summary data received was highly variable and dependent on the priority settings of each Argos message type (Table S2). Behavioral differences between tagged whales likely also affected the chance of a tagged whale being at the surface when a satellite was overhead. Transmission priorities were the same for ADB tags deployed on sperm whales in 2011 and 2013, allowing comparison. Behavior messages from these tags summarized an average of 62% of all qualifying dives made, while histogram messages summarized an average of 50% of the tracking period across both years. Tags which drifted for extended periods of time before recovery generated a higher rate of data return due to the uninterrupted transmissions while the tag was floating at the surface.

## 4 Discussion

### 4.1 Complications with attachment and release

Variability in attachment duration of the ADB tags was strongly influenced by the depth of tag penetration upon deployment (Table S1). The tag float and housing plate produce a substantial amount of hydrodynamic drag as the whale moves through the water, which acts to pull the tag out. Achieving full penetration, so that the housing plate is flush against the skin of the whale, not only allows more time for the housing to be fully extracted from the whale, but it also reduces the area of the tag exposed to hydrodynamic drag, thereby lengthening the attachment duration.

Recovery of the archived data required that the tag triggers a corrodible release wire in order to separate from its housing for subsequent recovery. This process created a number of challenges across generations of ADB tags. After having deployed 10 Generation‐1 tags in 2007, tags began releasing prematurely from their housings without having met any of the three release criteria. After recovery and evaluation of the released tags, testing on the remaining undeployed tags suggested that rotation of the tag in the housing could shear the corrodible link wire causing them to release early. Therefore, the diameter of the corrodible wire was doubled on tags deployed the following years and three small posts were added to the housing plate that fit into corresponding indentations in the bottom of the tag float to prevent rotation. These modifications appeared to be successful, as none of the tags deployed in 2008, or any following year, released early.

The tag float was not sufficiently buoyant to support both the tag and its attachment housing, so tags sank when their housings were shed from a whale prior to the scheduled release time. Tags were programmed to detect a lack of significant depth change over 24 hr to provide for tag release under such circumstances. With the exception of the epoxy casting issue in 2008, the cessation of satellite transmissions was assumed to indicate the tag and housing had been shed and sunk to the bottom. In 2011, only one of 11 Generation‐2 tags continued transmitting until the programmed release date, suggesting the others had been shed, and none released from their housings 24 hr after the last transmission as programmed. Subsequent laboratory testing revealed that cold temperature and lack of water flow for tags sitting on the bottom likely extended the time required to corrode the release wire beyond the preprogrammed duration current was allowed to pass through the wire (set to conserve battery power for recovery transmissions). This time restriction was removed for all future generations of tags and housing release and recovery rates dramatically improved. After 2011, there were four instances where tags were likely shed but did not release from their housings, but diagnostic information is limited in those cases to two tags that were subsequently recovered by beachgoers (>1 year later in both cases). We speculate these may have been the result of a mechanical impediment to the tag separating from the housing (i.e., laying among rocks or in mud), or some unknown tag failure.

Four tags deployed in 2015 remained attached to their housings for multiple days past the programmed release time before eventually separating from the housing. This issue was not observed in previous years where a number of tags reached their programmed release dates or with three other tags deployed in 2015. Two of the tags that were eventually recovered had a sticky substance on the float near the remains of the corrodible wire. A triple antibiotic ointment is applied to the blades prior to deployment, and we speculate the tight tag/tissue fit may have carried the oil‐based ointment up to the corrodible wire, limiting saltwater contact. Other recovered tags recorded voltage spikes after release was initiated, so there may have been a combination of factors affecting the release timing.

### 4.2 Applications for ADB tag data

The detailed data collected by ADB tags over periods of multiple weeks have the ability to expand on current research directions and create new opportunities. For example, recent cetacean research is progressing beyond characterization of behavior and into investigations of how behavior relates to foraging ecology, energetics and diving physiology (Goldbogen et al., 2015; Hazen, Friedlaender, & Goldbogen, 2015). Collection of such longer‐duration data records will dramatically improve temporal and spatial scales of observed trends and quantify individual variability.

Species distribution models for blue and fin whales have been developed with substantial success based on remotely sensed data like sea surface temperature and phytoplankton chlorophyll‐*a* concentration (Becker et al., 2016). In contrast, similar approaches with sperm whale data have been more variable, with some identifying direct relationships to environmental correlates, while others finding weak or negligible associations (Pirotta et al., 2011; Skov et al., 2008; Waring, Hamazaki, Sheehan, Wood, & Baker, 2001). The environmental cues driving sperm whale distribution continue to be elusive, likely due to their foraging at such deep depths. By monitoring the temperature profile of the water column and the spatial variation of foraging effort, ADB data may offer new insights regarding the water masses where the whales are foraging, and the scale on which they are searching for, and foraging on, sparsely distributed patchy prey (Palacios, Baumgartner, Laidre, & Gregr, 2013).

Characterization of cetacean responses to anthropogenic noise (military sonar, seismic surveys, vessel traffic, etc.) is a growing need (Nowacek et al., 2007; Soto et al., 2006; Southall et al., 2007) and currently the subject of substantial research (DeRuiter et al., 2013; Goldbogen, Calambokidis et al., 2013; Goldbogen, Southall et al., 2013; Harris et al., 2016). However, the experimental period of a majority of studies is limited by short‐attachment‐duration suction‐cup tags, preventing the collection of baseline (pre‐exposure) data on the subject animal, the duration of experimental exposures, and post‐experiment monitoring to estimate the duration of lasting effects (Nowacek et al., 2016). A longer‐duration data logger like the ADB tag would allow a better understanding of normal variations in whale behavior and the time scales over which they occur. Such information could be applied to experiments to better identify behavioral responses when they occur and better understand the implications of those responses (Nowacek et al., 2007). Meanwhile, the dive behavior summary messages transmitted via Argos could be used to monitor a whale's behavior in near‐real time for responses that exceed a behavioral threshold while an experiment is occurring.

While other transdermal tags (e.g., Argos satellite tags; Mate et al., 2007) and short‐duration data loggers will continue to be useful for a wide range of applications with large whales, intermediate‐duration archival tags like the ADB tag can bridge the gap between the two types of data. Many of the behavioral analyses developed for short‐duration, high‐resolution data loggers could be extended to ADB data while also accounting for spatial and temporal variability of those behaviors that previously could not be addressed. Conversely, behaviors and their corresponding movements described by the ADB data could be extended to better inform the more limited, but longer‐duration, data produced by transdermal tags. The result will be a dramatic improvement in our ability to study the behavior of large whales and the ecological mechanisms that drive it.

## Data Accessibility

The data used in this manuscript are reported in Tables S1 and S2.

## Conflict of Interest

None declared.
