# Supplementary material for: The development of an intermediate‐duration tag to characterize the diving behavior of large whales
Source: Ecol Evol. 2016 Dec 20;7(2):585–95. doi: 10.1002/ece3.2649 (PMC5243192; doi:10.1002/ece3.2649)
Supplement: Supplementary file 1 [file ECE3-7-585-s001.docx]

Supplemental Table S1: Deployment summary data for each ADB tag deployed.

| **Tag #** | **Year** | **Species** | **ADB tag type** | **Duration (days)** | **# Archived GPS Locations** | **# Archived Dives** | **Recovered?** |
| --- | --- | --- | --- | --- | --- | --- | --- |
| 2007_5963 | 2007 | Sperm | G1 | 9.7 | 447 | 362 | Yes |
| 2007_5841 | 2007 | Sperm | G1 | 2.6 | 123 | 126 | Yes |
| 2007_5843 | 2007 | Sperm | G1 | 0.6 | 31 | 39 | Yes |
| 2007_837 | 2007 | Sperm | G1 | 2.7 | 150 | 140 | Yes |
| 2007_5938 | 2007 | Sperm | G1 | 1.3 | N/A | N/A | No |
| 2007_5923 | 2007 | Sperm | G1 | 2.3 | N/A | N/A | No |
| 2007_829 | 2007 | Sperm | G1 | 0.8 | 42 | 31 | Yes |
| 2007_5883 | 2007 | Sperm | G1 | 13.4 | 729 | 547 | Yes |
| 2007_5910 | 2007 | Sperm | G1 | 7.3 | 439 | 289 | Yes |
| 2007_5922 | 2007 | Sperm | G1 | 0.7 | 38 | 31 | Yes |
| 2007_5921 | 2007 | Sperm | G1 | 2.0 | N/A | N/A | No^F^ |
| 2007_5882 | 2007 | Sperm | G1 | 34.5 | N/A | N/A | No^F^ |
| 2007_5660 | 2007 | Sperm | G1 | 1.2 | N/A | N/A | No^F^ |
| 2008_828 | 2008 | Sperm | G1 | 11.2 | N/A | N/A | No |
| 2008_837_1 | 2008 | Sperm | G1 | 9.5 | 281 | 348 | Yes |
| 2008_5841 | 2008 | Sperm | G1 | 1.5 | N/A | N/A | No |
| 2008_5878 | 2008 | Sperm | G1 | 1.4 | N/A | N/A | No |
| 2008_10835 | 2008 | Sperm | G1 | 17.8 | N/A | N/A | No |
| 2008_5660 | 2008 | Sperm | G1 | 1.6 | N/A | N/A | No |
| 2008_5843 | 2008 | Sperm | G1 | 0.5 | N/A | N/A | No |
| 2008_10822 | 2008 | Sperm | G1 | 15.5 | N/A | N/A | No |
| 2008_10828 | 2008 | Sperm | G1 | 2.5 | N/A | N/A | No |
| 2008_10832 | 2008 | Sperm | G1 | 2.2 | N/A | N/A | No |
| 2008_837_2 | 2008 | Sperm | G1 | 13.3 | N/A | N/A | No |
| 2008_23042 | 2008 | Sperm | G1 | 1.3 | N/A | N/A | No |
| 2008_10843 | 2008 | Sperm | G1 | 26.6 | 850 | 1183 | Yes |
| 2011_833 | 2011 | Sperm | G2 | 25.3 | N/A | N/A | No |
| 2011_838 | 2011 | Sperm | G2 | 20.5 | N/A | N/A | No |
| 2011_840 | 2011 | Sperm | G2 | 12.8 | N/A | N/A | No |
| 2011_4173 | 2011 | Sperm | G2 | 49.6 | 666 | 1111 | Yes |
| 2011_4177 | 2011 | Sperm | G2 | 34.8 | N/A | N/A | No |
| 2011_5640 | 2011 | Sperm | G2 | 17.9 | N/A | N/A | No |
| 2011_5644 | 2011 | Sperm | G2 | 29.9 | N/A | N/A | No |
| 2011_5654 | 2011 | Sperm | G2 | 34.2 | N/A | N/A | No |
| 2011_5685 | 2011 | Sperm | G2 | 28.4 | N/A | N/A | No |
| 2011_5701 | 2011 | Sperm | G2 | 24.4 | N/A | N/A | No |
| 2011_5838 | 2011 | Sperm | G2 | 9.6 | N/A | N/A | No |
| 2013_833 | 2013 | Sperm | G3 | 17.7 | 758 | 330 | Yes** |
| 2013_838 | 2013 | Sperm | G3 | 1.4 | N/A | N/A | No |
| 2013_840 | 2013 | Sperm | G3 | 24.9 | 1185 | 488 | Yes |
| 2013_4177 | 2013 | Sperm | G3 | 0.0 | N/A | N/A | No |
| 2013_5640 | 2013 | Sperm | G3 | 22.8 | 1355 | 671 | Yes |
| 2013_5654 | 2013 | Sperm | G3 | 18.6 | 220 | 424 | Yes |
| 2013_5701 | 2013 | Sperm | G3 | 15.6 | 769 | 390 | Yes |
| 2013_5790 | 2013 | Sperm | G3 | 0.6 | 59 | 27 | Yes |
| 2013_5838 | 2013 | Sperm | G3 | 16.7 | 758 | 335 | Yes |
| 2014_5644 | 2014 | Blue | G3 | 19.0 | 185 | 1068 | Yes |
| 2014_5650 | 2014 | Blue | G4 | 20.0 | 2297 | 2276 | Yes |
| 2014_5655 | 2014 | Blue | G3 | 19.8 | 799 | 2918 | Yes |
| 2014_5803 | 2014 | Blue | G4 | 18.3 | 2539 | 1832 | Yes |
| 2014_5685 | 2014 | Fin | G3 | 15.8 | 95 | 1140 | Yes |
| 2014_5790 | 2014 | Fin | G3 | 7.7 | N/A | N/A | No* |
| 2014_5838 | 2014 | Fin | G3 | 11.1 | N/A | N/A | Yes** |
| 2015_838 | 2015 | Blue | G4 | 25.9 | N/A | N/A | No |
| 2015_840 | 2015 | Blue | G3 | 24.8 | 1558 | 2075 | Yes |
| 2015_4177 | 2015 | Blue | G4 | 27.5 | 1480 | 2794 | Yes |
| 2015_5650 | 2015 | Blue | G4 | 28.9 | 2337 | 2280 | Yes |
| 2015_5644 | 2015 | Fin | G4 | 15.4 | N/A | N/A | No* |
| 2015_5654 | 2015 | Fin | G4 | 16.0 | 1,591 | 910 | Yes |
|  |  |  |  |  |  |  |  |
| *Released but not recovered | | |  |  |  |  |  |
| ** Shed and sunk then found on a beach | | | |  |  |  |  |
| ^F^ Fixed to the housing so recovery was impossible | | | |  |  |  |  |

Supplemental Table S2: Summary of data transmission settings and data received through Service Argos for each individual tag.

| **Tag #** | **Year** | **Species** | **ADB tag type** | **Duration (days)** | **# Argos GPS Locations** | **# Behavior Dives** | **# Histograms** | **Transmission Type Settings** | **Qualifying Dive Definition** |
| --- | --- | --- | --- | --- | --- | --- | --- | --- | --- |
| 2007_5963 | 2007 | Sperm | G1 | 9.7 | 195 | 164 | NA | Location: High, Histogram: NA, Behavior: Low | Depth > 10 m, Duration > 10 min |
| 2007_5841 | 2007 | Sperm | G1 | 2.6 | 38 | 12 | NA | Location: High, Histogram: NA, Behavior: Low | Depth > 10 m, Duration > 10 min |
| 2007_5843 | 2007 | Sperm | G1 | 0.6 | 20 | 13 | NA | Location: High, Histogram: NA, Behavior: Low | Depth > 10 m, Duration > 10 min |
| 2007_837 | 2007 | Sperm | G1 | 2.7 | 45 | 30 | NA | Location: High, Histogram: NA, Behavior: Low | Depth > 10 m, Duration > 10 min |
| 2007_5938 | 2007 | Sperm | G1 | 1.3 | 12 | 14 | NA | Location: High, Histogram: NA, Behavior: Low | Depth > 10 m, Duration > 10 min |
| 2007_5923 | 2007 | Sperm | G1 | 2.3 | 42 | 16 | NA | Location: High, Histogram: NA, Behavior: Low | Depth > 10 m, Duration > 10 min |
| 2007_829 | 2007 | Sperm | G1 | 0.8 | 25 | 11 | NA | Location: High, Histogram: NA, Behavior: Low | Depth > 10 m, Duration > 10 min |
| 2007_5883 | 2007 | Sperm | G1 | 13.4 | 211 | 160 | NA | Location: High, Histogram: NA, Behavior: Low | Depth > 10 m, Duration > 10 min |
| 2007_5910 | 2007 | Sperm | G1 | 7.3 | 96 | 46 | NA | Location: High, Histogram: NA, Behavior: Low | Depth > 10 m, Duration > 10 min |
| 2007_5922 | 2007 | Sperm | G1 | 0.7 | 8 | 0 | NA | Location: High, Histogram: NA, Behavior: Low | Depth > 10 m, Duration > 10 min |
| 2007_5921 | 2007 | Sperm | G1 | 2.0 | 17 | 19 | NA | Location: High, Histogram: NA, Behavior: Low | Depth > 10 m, Duration > 10 min |
| 2007_5882 | 2007 | Sperm | G1 | 34.5 | 347 | 316 | NA | Location: High, Histogram: NA, Behavior: Low | Depth > 10 m, Duration > 10 min |
| 2007_5660 | 2007 | Sperm | G1 | 1.2 | 18 | 10 | NA | Location: High, Histogram: NA, Behavior: Low | Depth > 10 m, Duration > 10 min |
| 2008_828 | 2008 | Sperm | G1 | 11.2 | 200 | 166 | NA | Location: High, Histogram: NA, Behavior: Low | Depth > 10 m, Duration > 10 min |
| 2008_837 | 2008 | Sperm | G1 | 9.5 | 249 | 226 | NA | Location: High, Histogram: NA, Behavior: Low | Depth > 10 m, Duration > 10 min |
| 2008_5841 | 2008 | Sperm | G1 | 1.5 | 20 | 26 | NA | Location: High, Histogram: NA, Behavior: Low | Depth > 10 m, Duration > 10 min |
| 2008_5878 | 2008 | Sperm | G1 | 1.4 | 8 | 2 | NA | Location: High, Histogram: NA, Behavior: Low | Depth > 10 m, Duration > 10 min |
| 2008_10835 | 2008 | Sperm | G1 | 17.8 | 188 | 157 | NA | Location: High, Histogram: NA, Behavior: Low | Depth > 10 m, Duration > 10 min |
| 2008_5660 | 2008 | Sperm | G1 | 1.6 | 23 | 16 | NA | Location: High, Histogram: NA, Behavior: Low | Depth > 10 m, Duration > 10 min |
| 2008_5843 | 2008 | Sperm | G1 | 0.5 | 9 | 9 | NA | Location: High, Histogram: NA, Behavior: Low | Depth > 10 m, Duration > 10 min |
| 2008_10822 | 2008 | Sperm | G1 | 15.5 | 293 | 259 | NA | Location: High, Histogram: NA, Behavior: Low | Depth > 10 m, Duration > 10 min |
| 2008_10828 | 2008 | Sperm | G1 | 2.5 | 49 | 22 | NA | Location: High, Histogram: NA, Behavior: Low | Depth > 10 m, Duration > 10 min |
| 2008_10832 | 2008 | Sperm | G1 | 2.2 | 32 | 16 | NA | Location: High, Histogram: NA, Behavior: Low | Depth > 10 m, Duration > 10 min |
| 2008_837 | 2008 | Sperm | G1 | 13.3 | 52 | 36 | NA | Location: High, Histogram: NA, Behavior: Low | Depth > 10 m, Duration > 10 min |
| 2008_23042 | 2008 | Sperm | G1 | 1.3 | 18 | 14 | NA | Location: High, Histogram: NA, Behavior: Low | Depth > 10 m, Duration > 10 min |
| 2008_10843 | 2008 | Sperm | G1 | 26.6 | 309 | 251 | NA | Location: High, Histogram: NA, Behavior: Low | Depth > 10 m, Duration > 10 min |
| 2011_833 | 2011 | Sperm | G2 | 25.3 | 135 | 488 | 172 | Location: Low, Histogram: Low, Behavior: High | Depth > 10 m, Duration > 10 min |
| 2011_838 | 2011 | Sperm | G2 | 20.5 | 97 | 416 | 122 | Location: Low, Histogram: Low, Behavior: High | Depth > 10 m, Duration > 10 min |
| 2011_840 | 2011 | Sperm | G2 | 12.8 | 13 | 229 | 76 | Location: Low, Histogram: Low, Behavior: High | Depth > 10 m, Duration > 10 min |
| 2011_4173 | 2011 | Sperm | G2 | 49.6 | 666 | 1111 | 422 | Location: Low, Histogram: Low, Behavior: High | Depth > 10 m, Duration > 10 min |
| 2011_4177 | 2011 | Sperm | G2 | 34.8 | 101 | 709 | 317 | Location: Low, Histogram: Low, Behavior: High | Depth > 10 m, Duration > 10 min |
| 2011_5640 | 2011 | Sperm | G2 | 17.9 | 29 | 245 | 78 | Location: Low, Histogram: Low, Behavior: High | Depth > 10 m, Duration > 10 min |
| 2011_5644 | 2011 | Sperm | G2 | 29.9 | 143 | 634 | 184 | Location: Low, Histogram: Low, Behavior: High | Depth > 10 m, Duration > 10 min |
| 2011_5654 | 2011 | Sperm | G2 | 34.2 | 125 | 625 | 190 | Location: Low, Histogram: Low, Behavior: High | Depth > 10 m, Duration > 10 min |
| 2011_5685 | 2011 | Sperm | G2 | 28.4 | 124 | 584 | 180 | Location: Low, Histogram: Low, Behavior: High | Depth > 10 m, Duration > 10 min |
| 2011_5701 | 2011 | Sperm | G2 | 24.4 | 126 | 501 | 165 | Location: Low, Histogram: Low, Behavior: High | Depth > 10 m, Duration > 10 min |
| 2011_5838 | 2011 | Sperm | G2 | 9.6 | 46 | 10 | 3 | Location: Low, Histogram:Low, Behavior:High | Depth > 10 m, Duration > 10 min |
| 2013_833 | 2013 | Sperm | G3 | 17.7 | 39 | 231 | 64 | Location: Low, Histogram: Low, Behavior: High | Depth > 10 m, Duration > 10 min |
| 2013_838 | 2013 | Sperm | G3 | 1.4 | 0 | 12 | 3 | Location: Low, Histogram: Low, Behavior: High | Depth > 10 m, Duration > 10 min |
| 2013_840 | 2013 | Sperm | G3 | 24.9 | 441 | 540 | 126 | Location: Low, Histogram: Low, Behavior: High | Depth > 10 m, Duration > 10 min |
| 2013_4177 | 2013 | Sperm | G3 | 0.0 | 0 | 0 | 0 | Location: Low, Histogram: Low, Behavior: High | Depth > 10 m, Duration > 10 min |
| 2013_5640 | 2013 | Sperm | G3 | 22.8 | 451 | 438 | 96 | Location: Low, Histogram: Low, Behavior: High | Depth > 10 m, Duration > 10 min |
| 2013_5654 | 2013 | Sperm | G3 | 18.6 | 213 | 261 | 119 | Location: Low, Histogram: Low, Behavior: High | Depth > 10 m, Duration > 10 min |
| 2013_5701** | 2013 | Sperm | G3 | 15.6 | 662 | 208 | 113 | Location: Low, Histogram: Low, Behavior: High | Depth > 10 m, Duration > 10 min |
| 2013_5790 | 2013 | Sperm | G3 | 0.6 | 34 | 10 | 4 | Location: Low, Histogram: Low, Behavior: High | Depth > 10 m, Duration > 10 min |
| 2013_5838 | 2013 | Sperm | G3 | 16.7 | 420 | 254 | 78 | Location: Low, Histogram: Low, Behavior: High | Depth > 10 m, Duration > 10 min |
| 2014_5644 | 2014 | Blue | G3 | 19.0 | 56 | 727 | 150 | Location: Low, Histogram: Low, Behavior: High | Depth > 10 m, Duration > 2 min |
| 2014_5655 | 2014 | Blue | G3 | 19.8 | 117 | 1504 | 133 | Location: Low, Histogram: Low, Behavior: High | Depth > 10 m, Duration > 2 min |
| 2015_840 | 2015 | Blue | G3 | 24.8 | 36 | 588 | 55 | Location: Low, Histogram: Low, Behavior: High | Depth > 10 m, Duration > 2 min |
| 2014_5650 | 2014 | Blue | G4 | 20.0 | 177 | 1554 | 175 | Location: Low, Histogram: Low, Behavior: High | Depth > 10 m, Duration > 2 min |
| 2014_5803* | 2014 | Blue | G4 | 18.3 | 356 | 2341 | 286 | Location: Low, Histogram: Low, Behavior: High | Depth > 10 m, Duration > 2 min |
| 2015_838 | 2015 | Blue | G4 | 25.9 | 74 | 2289 | 205 | Location: Low, Histogram: Low, Behavior: High | Depth > 10 m, Duration > 2 min |
| 2015_4177 | 2015 | Blue | G4 | 27.5 | 89 | 918 | 106 | Location: Low, Histogram: Low, Behavior: High | Depth > 10 m, Duration > 2 min |
| 2015_5650 | 2015 | Blue | G4 | 28.9 | 72 | 898 | 170 | Location: Low, Histogram: Low, Behavior: High | Depth > 10 m, Duration > 2 min |
| 2014_5685 | 2014 | Fin | G3 | 15.8 | 35 | 513 | 73 | Location: Low, Histogram :Low, Behavior: High | Depth > 10 m, Duration > 2 min |
| 2014_5790 | 2014 | Fin | G3 | 13.3 | 100 | 965 | 123 | Location: Low, Histogram :Low, Behavior: High | Depth > 10 m, Duration > 2 min |
| 2014_5838 | 2014 | Fin | G3 | 4.9 | 14 | 290 | 37 | Location: Low, Histogram: Low, Behavior: High | Depth > 10 m, Duration > 2 min |
| 2015_5644** | 2015 | Fin | G4 | 15.4 | 671 | 1359 | 163 | Location: Low, Histogram: Low, Behavior: High | Depth > 10 m, Duration > 2 min |
| 2015_5654 | 2015 | Fin | G4 | 16.0 | 53 | 974 | 119 | Location: Low, Histogram: Low, Behavior: High | Depth > 10 m, Duration > 2 min |
| *Tag transmitted while floating at the surface for 15 d before recovery | | | | | |  |  |  |  |
| ** Tag released too far offshore for recovery but transmitted while floating at the surface until batteries were exhausted (float time = 28.4 d (2013_5701) and 24.3 d (2015_5644) | | | | | | | | |  |
